# Blockade of RyRs in the ER Attenuates 6-OHDA-Induced Calcium Overload, Cellular Hypo-Excitability and Apoptosis in Dopaminergic Neurons

**DOI:** 10.3389/fncel.2017.00052

**Published:** 2017-03-03

**Authors:** Lu Huang, Ying Xue, DaYun Feng, RuiXin Yang, Tiejian Nie, Gang Zhu, Kai Tao, GuoDong Gao, Qian Yang

**Affiliations:** Department of Neurosurgery, Tangdu Hospital, Fourth Military Medical UniversityXi’an, China

**Keywords:** Parkinson’s disease, calcium, ER stress, Inositol 1, 4, 5-triphosphate receptors, ryanodine receptors, 6-hydroxydopamine

## Abstract

Calcium (Ca^2+^) dyshomeostasis induced by endoplasmic reticulum (ER) stress is an important molecular mechanism of selective dopaminergic (DA) neuron loss in Parkinson’s disease (PD). Inositol 1,4,5-triphosphate receptors (IP_3_Rs) and ryanodine receptors (RyRs), which are located on the ER surface, are the main endogenous Ca^2+^ release channels and play crucial roles in regulating Ca^2+^ homeostasis. However, the roles of these endogenous Ca^2+^ release channels in PD and their effects on the function and survival of DA neurons remain unknown. In this study, using a 6-hydroxydopamine (6-OHDA)-induced *in vitro* PD model (SN4741 Cell line), we found that 6-OHDA significantly increased cytoplasmic Ca^2+^ levels ([Ca2+]_i_), which was attenuated by pretreatment with 4-phenyl butyric acid (4-PBA; an ER stress inhibitor) or ryanodine (a RyRs blocker). In addition, in acute midbrain slices of male Sprague-Dawley rats, we found that 6-OHDA reduced the spike number and rheobase of DA neurons, which were also reversed by pretreatment with 4-PBA and ryanodine. TUNEL staining and MTT assays also showed that 4-PBA and ryanodine obviously alleviated 6-OHDA-induced cell apoptosis and devitalization. Interestingly, a IP_3_Rs blocker had little effect on the above 6-OHDA-induced neurotoxicity in DA neurons. In conclusion, our findings provide evidence of the different roles of IP_3_Rs and RyRs in the regulation of endogenous Ca^2+^ homeostasis, neuronal excitability, and viability in DA neurons, and suggest a potential therapeutic strategy for PD by inhibiting the RyRs Ca^2+^ channels in the ER.

## Introduction

Parkinson’s disease (PD), which is initially characterized by the loss of dopaminergic (DA) neurons in the SNc, is the second most common neurodegenerative disease. The molecular mechanisms of selective DA neuronal loss include genetic mutations, mitochondrial dysfunction, oxidative stress, and ER stress. Reports have indicated the ER as a crucial participant in the pathology of PD (Mercado et al., 2016). The accumulation of unfolded/misfolded proteins can induce ER stress, which is either physiologically eliminated by the unfolded protein response (UPR) or pathologically induces intracellular Ca^2+^ dyshomeostasis and endoplasmic reticulum associated death (ERAD; Mahdi et al., 2016).

As an endogenous store for Ca^2+^, the ER is vital in intracellular Ca^2+^ regulation. Cytoplasmic Ca^2+^ levels are mostly affected by ER-localized Ca^2+^ channels, including IP_3_Rs, RyRs, and the sarco/endoplasmic reticulum Ca^2+^-ATPase (SERCA; Durante et al., 2004). IP_3_Rs and RyRs are the main endogenous Ca^2+^ release channels, which mediate Ca^2+^ release from the ER lumen into the cytoplasm in response to extracellular stimuli. IP_3_Rs and RyRs are structurally and functionally analogous, but they also exhibit key differences. RyRs and IP_3_Rs reside in different locations and microenvironments (Carrasco et al., 2004). Additionally, the opening of IP_3_Rs is enhanced (0.5–1 μM) and inhibited (>1 μM) by modest increases in Ca^2+^ concentrations compared with normal cytoplasmic Ca^2+^ concentrations (∼100 nM; Bootman and Lipp, 1999), whereas the opening of RyRs is generally activated and inhibited by higher concentrations of Ca^2+^ (activation at 1–10 μM; inhibition at >10 μM; Ogawa and Murayama, 1995; Franzini-Armstrong and Protasi, 1997; Mikoshiba, 2007). Currently, how these channels regulate Ca^2+^ homeostasis in response to neurotoxic stimuli in PD remains unclear.

Ca^2+^, as a second messenger, is important for physiologically maintaining cellular excitability, protein structure and neurotransmitter release in neurons (Li et al., 2014). The Ca^2+^ content in the ER and cytoplasm is very important for neuron-fate decisions. The high concentration of Ca^2+^ in the ER lumen leads cells to undergo apoptosis, and maintaining low levels of Ca^2+^ is therefore protective against apoptosis. However, extremely low Ca^2+^ levels lead to ER stress via the accumulation of misfolded proteins in the ER (Decuypere et al., 2011; Mahdi et al., 2016). In addition, neuronal survival is also compromised when cytoplasmic Ca^2+^ levels fall beyond the physiological range in the cytosol (Oh et al., 2009; Xue et al., 2012).

Dopaminergic neurons are unique with regard to their autonomic excitability and selective dependence on Ca^2+^ channels rather than Na^+^ channels for action potential generation (Grobaski et al., 1997; Bonci et al., 1998; Ping and Shepard, 1999). Intracellular injection of high Ca^2+^ concentrations was reported to increase burst firing, while injection of the Ca^2+^ chelator EGTA reduced the depolarization that initiates the bursting, thus also greatly reducing the bursting of midbrain DA neurons. Although Ca^2+^ plays such a vital role in the excitability of DA neuron, it remains unclear which endogenous channels are related to the dysfunction of DA neurons and whether there is any connection between endogenous Ca^2+^ channels and alterations in DA neuronal excitability. However, the relationship between the specialized Ca^2+^-dependent excitability of DA neurons and ER Ca^2+^ channels remains unknown.

In this study, we investigated the roles of ER stress and the two endogenous Ca^2+^ release channels (IP_3_Rs and RyRs) in Ca^2+^ homeostasis and viability in SN4741 cells since it is a DA neuronal cell line of embryonic SN origin which is widely accepted more accessible to molecular analysis and used as an *in vitro* model system for study SNc DA neurons (Son et al., 1999). We also investigated cellular excitability in a 6-OHDA-induced PD model in midbrain slices. Our data demonstrated the different roles of IP_3_Rs and RyRs in 6-OHDA-mediated neurotoxicity in DA neurons and suggested a potential therapeutic strategy for PD through the inhibition of RyRs Ca^2+^ channels in ER.

## Experimental Procedures

### Preparation of Acute Midbrain Slices

All animal work was performed according to the guidelines of the Institutional Animal Care and Use Committee at the Fourth Military Medical University. Carefully prepared slices containing the SNc (300 μm thick) were separated from male Sprague-Dawley rats (12–14 days of age) as described previously (Xue et al., 2012). Briefly, animals were sacrificed, and the brain was rapidly removed and immersed in ice-cold modified ACSF [in mM: 11 glucose, 126 NaCl, 18 NaHCO_3_, 1.2 NaH_2_PO_4_, 2.5 KCl, 2.4 CaCl_2_, 1.3 MgCl_2_; bubbled with a gas mixture (95% O_2_/5% CO_2_; pH 7.4)] for more than 30 min. Then, the midbrain was blocked in a coronal plane, fixed to the stage of a VT1000P vibratome (LEICA, Germany) and sliced. We maintained slices at room temperature for 1 h before experimentation.

### Drugs and Chemicals

The following chemical agents were used in the experiment: 6-OHDA (1 μM, MH116, Sigma-Aldrich), 4-PBA (2 mM, sc-200652, Santa Cruz), BAPTA-AM (10 μM, A1076, Sigma-Aldrich), RY (100 μM, SML1106, Sigma-Aldrich), and Xes (800 nM, X2628, Sigma-Aldrich).

### Electrophysiological Recording

A 3–6 MΩ micropipette, pulled by a P-97 (Sutter Instrument, USA) puller, was used to patch the neurons. The intracellular solution contained (in mM) 125 K-gluconate, 1.2 MgCl_2_, 10 HEPES, 1 EGTA, 0.3 CaCl_2_, 0.3 Na-GTP, and 2.1 Mg-ATP, pH 7.3 using NaOH (290–300 mOsm). Experiments were discarded if the access resistance crossed 25 MΩ. The procedures for insulation of DA neurons were performed according to previous reports (Durante et al., 2004). Whole-cell patch-clamp recordings were obtained from the target neurons in the SNc. The DA neurons were characterized by regular and slow autonomous firing (2–4 Hz) and were clearly different from the GABAergic neurons in the SNc.

Data were recorded by filtering at 3 kHz and digitizing at 10 kHz at room temperature (20–24°C) and analyzed in pCLAMP 10.2 software (Axon Instruments Inc., USA). We used current-clamp recordings to evaluate evoked action potentials in the experiments in which we normalized the resting membrane potential to –60 mV in prepared slices. Neurons were considered to be ‘stable’ and included in the data analysis when their input resistance oscillated by no more than 20%. The current step was set from –200 to 400 pA, with an increment of 25 pA, a duration of 1 s, and a 10 s interpulse interval. Rheobase current amplitude was the minimal injected current capable of evoking an action potential.

### Cell Culture

SN4741 cells were derived from the embryonic SNc of a transgenic mouse. We cultured the cells at 33°C and 5% CO_2_ in high-glucose Dulbecco’s minimum essential medium (DMEM) supplemented with 1% D-glucose, 2 mM L-glutamine, and 10% heat-inactivated fetal bovine serum (FBS).

### Calcium Imaging

Free Ca^2+^ was measured by fluorescence imaging using the Ca^2+^ indicator dye fluo-3AM (Beyotime, China) based on previously detailed methods (Guatimosim et al., 2011). SN4741 cells were cultured in the 3.5 mm plates (2 × 10^5^ cells/well) for 24 h, then cells were incubated with 5 μM fluo-3AM at 33°C for 30 min. Cells were then washed twice with fresh buffer (in mM; 132 NaCl, 5 KCl, 10 dextrose, 10 HEPES, and 1.05 MgCl_2_, pH 7.4). The dye-loaded cells were incubated in fresh buffer or pretreated with 4-PBA, RY or Xes for another 20 min before imaging. Before exposed to 6-OHDA (fresh buffer was applied for control group), the cells were scanned for 3 min to obtain a basal fluorescence intensity level of intracellular Ca^2+^ (F_0_) and another 27 min under the treatments to obtain the real-time fluorescence intensity (F), the ratio was *F*/*F*_0_. Cells were imaged with a laser scanning confocal microscope (A1; Nikon, Japan). The image was recorded every 30 s (HV 45, Laser 0.45, pinhole 1.2, ROI area 130) and the fluorescence intensity was obtained by the Nikon A1 analysis system. Data were presented as the ratio of fluo-3AM fluorescence intensity (*R* = *F*/*F*_0_, where *F* and *F*_0_ are the current and baseline fluorescence values, respectively) which was normalized to control.

### Mitochondrial Staining

Mitochondrial staining was performed according to the manufacturer’s instructions. SN4741 cells were cultured on in the 3.5 mm plates (2 × 10^5^ cells/well) for 24 h. After treatment, the cells were incubated with 10 nM MitoTracker Red CMXRos (Life Technologies) for 20 min at 33°C. Mitochondria were imaged under a laser scanning confocal microscope (A1; Nikon, Japan). Quantification of the mitochondria morphology was performed as described previously (Koopman et al., 2008).

### ROS Detection

CM-H2DCFDA (Life Technologies) was used in the measurement of the ROS production. SN4741 cells were cultured on in the 3.5 mm plates (2 × 10^5^ cells/well) for 24 h. After designed treatment, cells were incubated with 5 μM dye for 30 min at 33°C. Cells were washed three times with prewarmed PBS at 33°C to remove excess dye. ROS-positive cells were visualized via laser scanning confocal microscopy (A1; Nikon, Japan) and counted using Image J software (the whole cells were accounted according to bright-field and the positive cells were calculated according to ROS staining. The ratio is the specific value of positive cells and whole cells.

### TUNEL Staining

The Cell Death Detection Kit (11684795910, Roche) was utilized to measure cell apoptosis. SN4741 cells were pretreated with 4-PBA, ryanodine, or Xes for 20 min before exposure to 6-OHDA for 2 h. The cells were then fixed with 4% paraformaldehyde, and the TUNEL reaction was performed according to the manufacturer’s protocol. TUNEL-positive cells were visualized via laser scanning confocal microscopy (A1; Nikon, Japan) and counted using Image J software (the whole cells were accounted according to DAPI staining) and the positive cells were calculated according to TUNEL staining. The ratio is the specific value of positive cells and whole cells.

### MTT Assay

SN4741 cells (6 × 10^5^ cells/ml) were seeded into a 96-well polystyrene tissue culture plate and incubated for 24 h at 37°C and 5% CO_2_. After treatment, the culture medium was replaced, and 20 μl of MTT solution (5 mg/ml in PBS) was added to every well. Then, 100 μl of dimethyl sulfoxide (DMSO) was added to each well for 4 h, after which the plate was read at 490 nm. All assays were performed in ten replicates per dose.

### Statistical Analysis

Patch clamp data obtained from all treatments were analyzed using pClamp 10.2 software (Axon Instruments Inc., USA) and Origin 8.0 software (Origin Lab, Northampton, MA, USA). The fluorescence intensity of Calcium imaging results from cells with various treatments was obtained using a Nikon A1 analysis system and analyzed with graphpad prism software. Two-way ANOVA was used to compare the differences of changes in calcium level as we select time as row factor. Data were presented as the means ± S.E.M. Statistical analysis of the results was performed using paired *t*-tests or one-way ANOVA for the current data (rheobase) and two-way ANOVA for spike number vs. injection current(the row factor was injection current) and calcium imaging fluorescence intensity vs. time(the row factor was time). Statistical significance was two-sided, and the level of statistical significance was set at *P* < 0.05.

## Results

### Effect of 4-PBA on Ca^2+^ Dyshomeostasis Induced by 6-OHDA in DA Neurons

**FIGURE 1:**
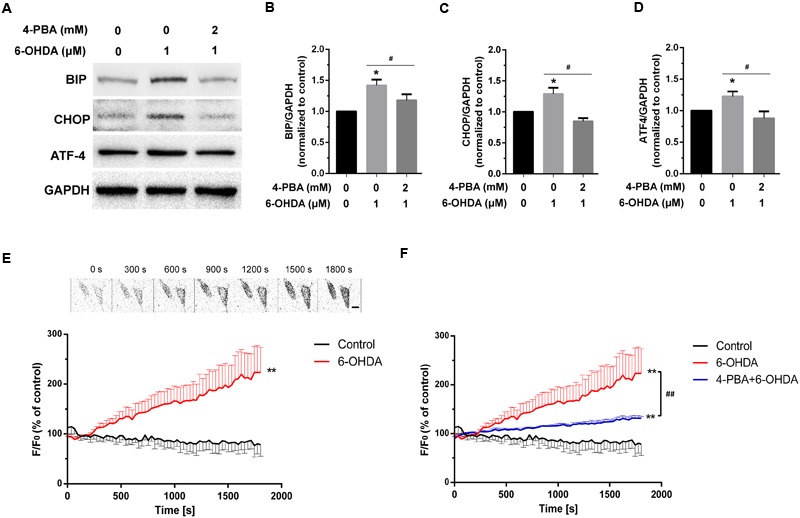
**The effect of 4-PBA on 6-OHDA-induced Ca^2+^ dyshomeostasis in DA neurons.** Western blot and analysis of SN4741 cells treated with 6-OHDA (1 μM) without or with 4-PBA (2 mM) pretreatment for 20 min **(A–D)**. Plots of the fluorescence ratio in DA neurons treated with 6-OHDA (1 μM) without **(E)** or with **(F)** 4-PBA (2 mM) pretreatment for 20 min. The sketch map in **(E)** was just a series of screenshot of one cell in the group attempted to offer direct-viewing impression of our data. Data are shown as mean ± SEM (*n* = 10). The Plots of the fluorescence ratio was analyzed using two-way ANOVA and the row factor was time, while the Western blot was analyzed through gray-scale value. BIP: Control, 1(set as standard); 6-OHDA 1.42 ± 0.09; 4-PBA+6-OHDA, 1.18 ± 0.10. ATF4: Control, 1(set as standard); 6-OHDA 1.23 ± 0.08; 4-PBA+6-OHDA, 0.88 ± 0.11. CHOP: Control, 1(set as standard); 6-OHDA 1.28 ± 0.10; 4-PBA+6-OHDA, 0.85 ± 0.05. ^∗^*P* < 0.05 and ^∗∗^*P* < 0.01 vs. the control group; ^#^*P* < 0.05 and ^##^*P* < 0.01 in the chosen groups. Scale bar = 25 μm.

**FIGURE 2:**
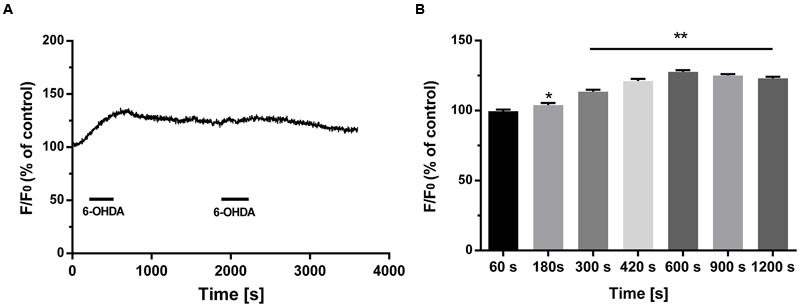
**The irreversible effect of 6-OHDA on cytoplasmic Ca^2+^ in DA neurons.** Plots of the fluorescence ratio in DA neurons treated with 6-OHDA (1 μM) for 5 min followed by washout for another 10 min **(A,B)**. Data are shown as mean ± SEM (*n* = 10). The fluorescence ratio was analyzed via one-way ANOVA. ^∗^*P* < 0.05 and ^∗∗^*P* < 0.01 vs. the control group.

Previous studies have indicated that extracellular Ca^2+^ plays an important role in increased cytoplasmic Ca^2+^ levels (Qu et al., 2014). However, whether intracellular Ca^2+^ is involved in 6-OHDA-induced cytoplasmic Ca^2+^ dyshomeostasis is still unclear. Western blot supported that treatment of 6-OHDA (1 μM) can induced ER stress and this effect can be reversed by pretreatment of ER stress inhibitor 4-PBA (2 mM, 20 min; **Figures 1B–D**, one-way ANOVA). Thus, we treated SN4741 cells with fresh buffer solution lacking Ca^2+^ (in mM; 132 NaCl, 5 KCl, 10 dextrose, 10 HEPES, and 1.05 MgCl_2_, adjusted to pH 7.4 using 1 M NaOH) and then measured the fluorescence in the presence or absence of 6-OHDA (1 μM) for 30 min. Fluorescence staining analysis revealed that 6-OHDA produced a significant [Ca^2+^]_i_ increase in the absence of extracellular Ca^2+^ (**Figure 1E**, *n* = 10, two-way ANOVA, the row factor was time). Next, we tested whether the increase in [Ca^2+^]_i_ was related to the ER Ca^2+^ stores. Pretreated cells with the ER stress inhibitor 4-PBA (2 mM, 20 min) reversed the 6-OHDA-induced [Ca^2+^]_i_ increase (**Figure 1F**). Interestingly, the change of [Ca^2+^]_i_ did not recover, even after a 20 min drug washout (**Figures 2A,B**, *n* = 10, one-way ANOVA of 2F).

### The Different Effects of IP_3_Rs and RyRs on Ca^2+^ Overload Induced by 6-OHDA in DA Neurons

**FIGURE 3:**
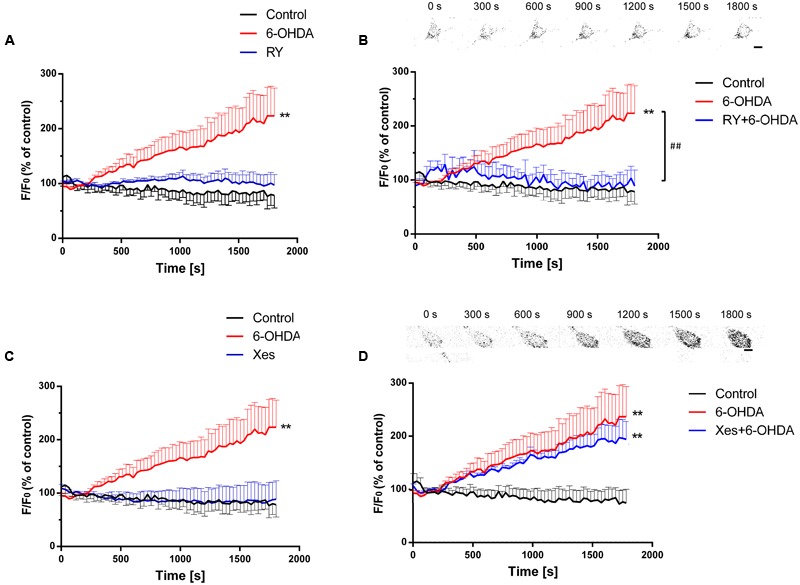
**The different effects of IP_3_Rs and RyRs on 6-OHDA-induced Ca^2+^ overload in DA neurons.** Plots of the fluorescence ratio in DA neurons treated with RY (100 μM) or 6-OHDA (1 μM) alone **(A)**, pretreated with RY (100 μM) for 20 min and then with 6-OHDA (1 μM) **(B)**, treated with Xes (800 nM) or 6-OHDA (1 μM) alone **(C)**, or pretreated with Xes (800 nM) for 20 min and then with 6-OHDA (1 μM) **(D)**. The sketch map in **(B,D)** was just a series of screenshot of one cell in the group attempted to offer direct-viewing impression of our data. Data are shown as mean ± SEM (*n* = 10). The Plots of the fluorescence ratio was analyzed via two-way ANOVA, the row factor was time ^∗∗^*P* < 0.01 vs. the control group and ^##^*P* < 0.01 in the chosen groups. Scale bar = 25 μm.

Ca^2+^ release from the ER mainly occurs via two Ca^2+^ channels: IP_3_Rs and RyRs (Krebs et al., 2015). Here, we explored the roles of these Ca^2+^ channels in a 6-OHDA-induced PD model *in vitro*. SN4741 cells were pretreated with the RyRs inhibitor ryanodine (RY, 100 μM) or the IP_3_Rs inhibitor Xes (800 nM) for 20 min followed by incubation with 6-OHDA (1 μM). Calcium imaging revealed that 6-OHDA significant increased [Ca^2+^]_i_ levels (*P* < 0.01) and that treatment with RY alone failed to induce significant changes in [Ca^2+^]_i_ levels in SN4741 cells (**Figure 3A**, *n* = 10, two-way ANOVA, the row factor was time). However, pretreatment with RY markedly decreased the 6-OHDA-induced [Ca^2+^]_i_ increase in SN4741 cells (*P* < 0.01; **Figure 3B**, *n* = 10, two-way ANOVA, the row factor was time). In contrast, treating with Xes alone did not alter [Ca^2+^]_i_ levels in SN4741 cells (**Figure 3C**, *n* = 10, two-way ANOVA, the row factor was time), and pretreatment with Xes also showed little effect on the 6-OHDA-induced [Ca^2+^]_i_ increase (**Figure 3D**, *n* = 10, two-way ANOVA, the row factor was time). These data suggest that the RyRs channel rather than the IP_3_Rs channel primarily mediates the 6-OHDA-induced Ca^2+^ overload in DA neurons.

### The Effects of 4-PBA on 6-OHDA-Induced Hypo-Excitability in DA Neurons of the SNc

**FIGURE 4:**
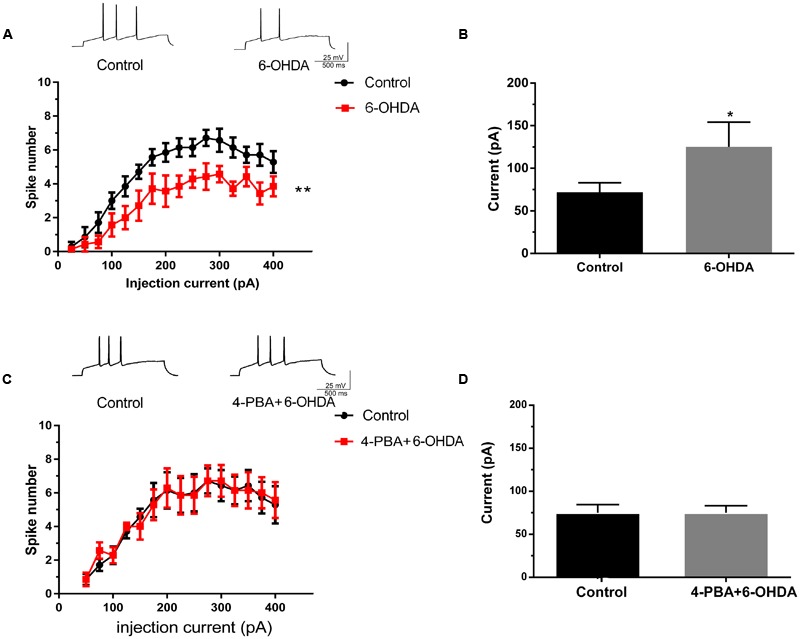
**The effects of 4-PBA on the 6-OHDA-induced hypo-excitability of DA neurons in the SNc.** Plots of spike number vs. injection current and average current of the rheobase in DA neurons in SNc slices treated with 6-OHDA (1 μM) alone **(A,B)** or with 4-PBA (2 mM) pretreatment for 20 min **(C,D)**. Data are shown as mean ± SEM (*n* = 10). The Plots of spike number vs. injection current was analyzed via two-way ANOVA, the row factor is injection current while the average current of the rheobase was analyzed via *t*-test. Rheobase: Control, 67.93 ± 23.47 pA; 6-OHDA 147.5 ± 48.92 pA; Control, 68.75 ± 19.24 pA; 4-PBA+6-OHDA, 71.88 ± 22.17 pA; ^∗^*P* < 0.05 and ^∗∗^*P* < 0.01 vs. the control group. The sketch map was recorded at the injection current was 100 pA.

Acute separated midbrain slices were used to assess the physiological function of DA neurons in the SNc. Following incubation with 6-OHDA (1 μM) for 10 min, the electrical activity of DA neurons was recorded. The results showed that the spike number vs. injection current was obviously decreased while the average rheobase current was significantly increased, which represented the excitability level of the neurons was reduced. (**Figures 4A,B**, *n* = 10, two-way ANOVA of spike number, the row factor was injection current, *t*-test of rheobase). In addition, pretreatment with the ER stress inhibitor 4-PBA significantly reversed the 6-OHDA-induced decrease in spike number and the rheobase, which indicated that these changes in neuronal excitability might be closely related to ER stress in DA neurons (**Figures 4C,D** and Supplementary Figures 1A, 2A). Our results demonstrated that 4-PBA can alleviate 6-OHDA-induced hypo-excitability in DA neurons in the SNc.

### The Different Effects of IP3Rs and RyRs on the 6-OHDA-Induced Hypo-Excitability of DA Neurons in the SNc

**FIGURE 5:**
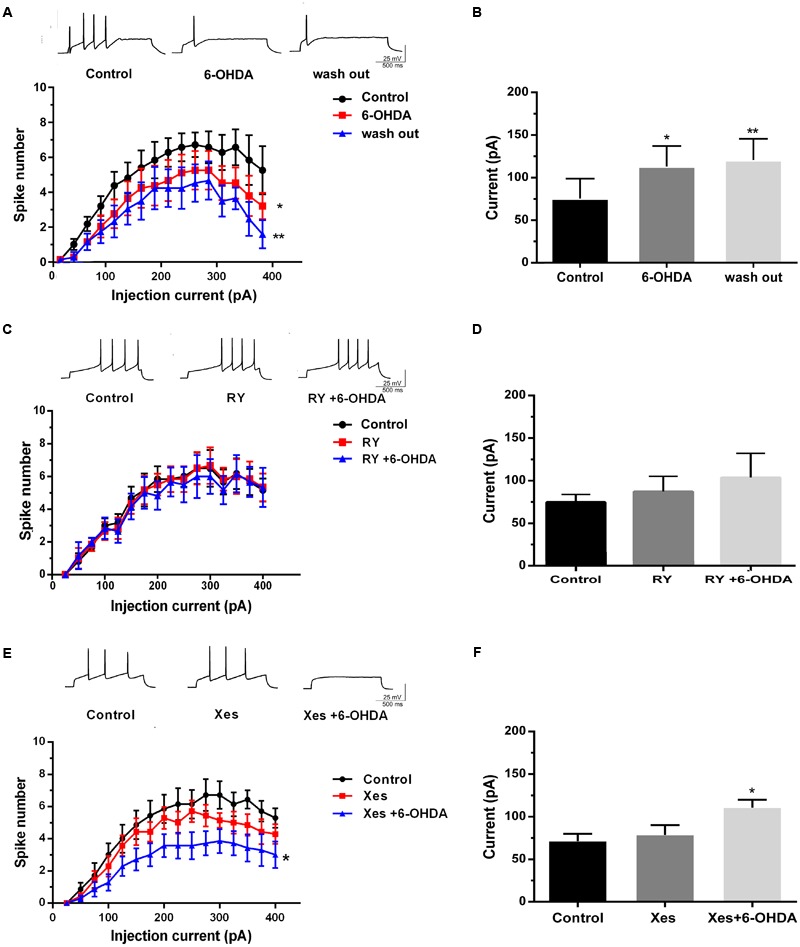
**The different effects of IP_3_Rs and RyRs channels on the 6-OHDA-induced hypo-excitability of DA neurons in the SNc.** Plots of spike number vs. injection current and average current of the rheobase in DA neurons in SNc slices treated with 6-OHDA (1 μM) and incubated in ACSF for another 10 min **(A,B)**, pretreated with RY (100 μM) for 20 min followed by 6-OHDA (1 μM) **(C,D)**, and pretreated with Xes (800 nM) for 20 min followed by 6-OHDA (1 μM) **(E,F)**. Data are shown as mean ± SEM (*n* = 10). The Plots of spike number vs. injection current was analyzed via two-way ANOVA, the row factor is injection current while the average current of the rheobase was analyzed via one-way ANOVA. Rheobase: Control, 71.43 ± 26.37 pA; 6-OHDA, 107.14 ± 27.92 pA; wash out, 122.49 ± 30.41 pA; Control, 71.88 ± 9.43 pA; RY, 87.78 ± 27.14 pA; RY+6-OHDA, 91.72 ± 32.17 pA; Control, 67.81 ± 10.01 pA; Xes, 78 ± 2.15 pA; Xes+6-OHDA, 113 ± 8.21 pA; ^∗^*P* < 0.05 and ^∗∗^*P* < 0.01 vs. the control group. The sketch map was recorded at the injection current was 100 pA.

To identify whether the neurotoxicity of 6-OHDA is transient or irreversible, we investigated neuronal excitability using a 10 min drug washout by ACSF following 6-OHDA stimulation. The results showed that drug washout failed to recover the decrease in spike number and the average rheobase current induced by 6-OHDA in DA neurons in SNc slices (**Figures 5A,B**, *n* = 10, two-way ANOVA of spike number, the row factor was injection current, one-way ANOVA of rheobase; Supplementary Figures 1B, 2B). We pretreated with RY (100 μM, 20 min) and Xes (800 nM) to verify the roles of the two Ca^2+^-release channels in 6-OHDA-induced neuronal hypo-excitability. The results showed that neither RY nor Xes treatment alone could alter the spike number and rheobase in DA neurons. However, RY pretreatment significantly blocked the inhibition of neuronal excitability induced by 6-OHDA in DA neurons (**Figures 5C,D**, *n* = 10, two-way ANOVA of spike number, the row factor was injection current, one-way ANOVA of rheobase; Supplementary Figures 1C, 2C), but this effect was not significant in Xes pretreatment (**Figures 5E,F**, *n* = 10, two-way ANOVA of spike number, the row factor was injection current, one-way ANOVA of rheobase; Supplementary Figures 1D, 2D). These data imply that RyRs, rather than IP_3_Rs, primarily mediate the 6-OHDA-induced hypo-excitability in DA neurons.

### Blockade of ER Stress and Ca^2+^ Overload Protected against Mitochondrial Dysfunction Induced by 6-OHDA

**FIGURE 6:**
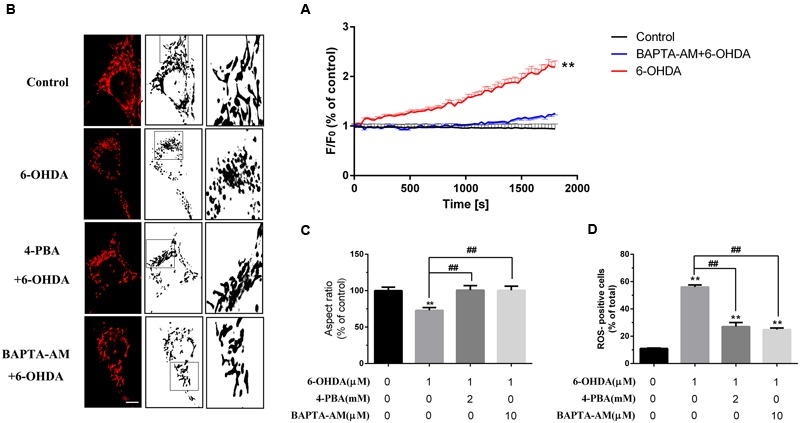
**Blockade of ER stress and Ca^2+^ overload protected against mitochondrial dysfunction induced by 6-OHDA.** Plots of the fluorescence ratio in DA neurons treated with 6-OHDA (1 μM) without or with pretreatment of BAPTA-AM (10 μM) for 20 min **(A)**. Mitochondrial morphology and analysis of SN4741 cells treated with 6-0HDA (1 μm) without or with pretreatment of BAPTA-AM (10 μM) or 4-PBA (2 mM) for 20 min **(B,C)**. ROS of SN4741 cells treated with 6-0HDA (1 μm) without or with pretreatment of BAPTA-AM (10 μM) or 4-PBA (2 mM) for 20 min **(D)**. Data are shown as mean ± SEM (*n* = 7). The Plots of the fluorescence ratio was analyzed using two-way ANOVA and the row factor was time, while the mitochondrial morphology was analyzed through Aspect ratio and ROS through positive cells ratio via one-way ANOVA. Aspect ratio: Control, 100% (set as standard); 6-OHDA, 72.97 ± 3.83%; 4-PBA+6-OHDA, 100.17 ± 6.22%; BAPTA-AM+6-OHDA, 100.04 ± 5.73%. ROS: Control, 11.02 ± 0.30%; 6-OHDA, 56.04 ± 1.49%; 4-PBA+6-OHDA, 27.04 ± 2.98%; BAPTA-AM+6-OHDA, 28.70 ± 1.20%. ^∗∗^*P* < 0.01 vs. the control group and ^##^*P* < 0.01 in the chosen groups. Scale bar = 20 μm.

Several studies have confirmed that 6-OHDA can induce oxidative stress *in vivo* as well as *in vitro*. Some studies implied that the dysfunction of mitochondrial may closely associated to ER stress (Li et al., 2014). Our data showed that BAPTA-AM can eliminate the increase of [Ca^2+^]_i_ induced by 6-OHDA (**Figure 6A**) and pretreatment of ER stress inhibitor 4-PBA (2 mM) or blockade of [Ca^2+^]_i_ overload by BAPTA-AM (10 μM) can protect the mitochondrial from 6-OHDA induced dysfunction(**Figures 6B,C**) as well as ROS generation (**Figure 6D**). These data implies that calcium may serves as a critical mediator linking changes in ER homeostasis to the onset of mitochondrial dysfunction.

### The Protective Effects of ER Stress Inhibition and RyRs Blockade on DA Neuronal Viability in Response to 6-OHDA

**FIGURE 7:**
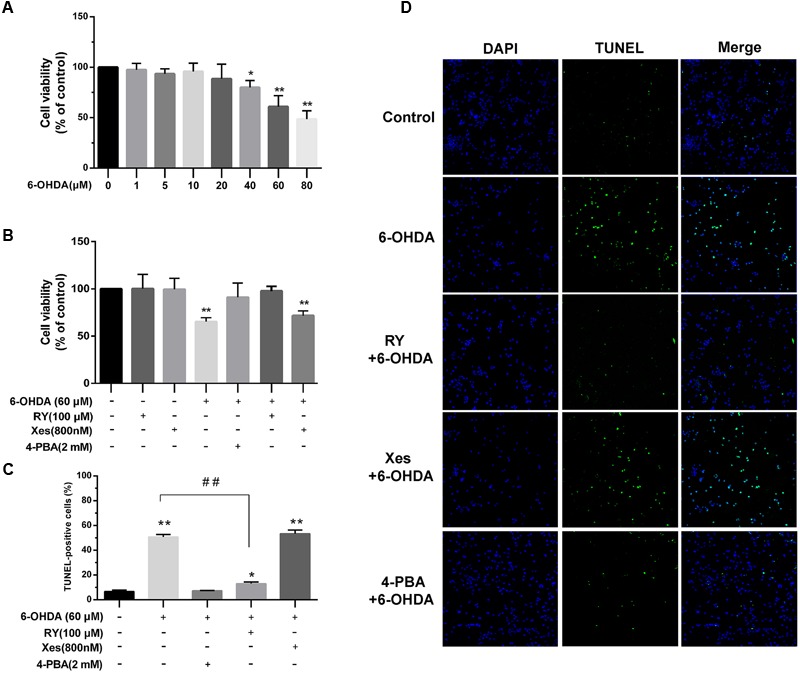
**The protective effects of ER stress inhibition and RyRs blockade on the viability of DA neurons treated with 6-OHDA. (A,B)** Cell viability was measured using MTT assays. ^∗^*P* < 0.05 and ^∗∗^*P* < 0.01 vs. the control group. **(C)** Cell apoptosis was analyzed by TUNEL staining. **(D)** Images of TUNEL staining Green: TUNEL-positive cells, Blue: DAPI. ^∗^*P* < 0.05 and ^∗∗^*P* < 0.01 vs. the control group. ^##^*P* < 0.01 vs. the indicated groups. Data are expressed as mean ± SEM (*n* = 5). The cell viability, cell apoptosis was analyzed via one-way ANOVA. Cell viability: Control, 100% (set as standard); 6-OHDA (1 μM), 97.6 ± 6.24%; 6-OHDA (5 μM), 93.46 ± 4.98%; 6-OHDA (10 μM), 95.81 ± 8.24%; 6-OHDA (20 μM), 88.58 ± 14.4%; 6-OHDA (40 μM), 79.99 ± 6.96%; 6-OHDA (60 μM), 60.96 ± 10.85%; 6-OHDA (80 μM), 48.52 ± 8.27%. Cell viability: Control, 100% (set as standard); RY 100.24 ± 15.25%; Xes, 99.51 ± 14.38%; 6-OHDA, 65.34 ± 4.21%; 4-PBA+6-OHDA, 91.01 ± 15.78%; RY+6-OHDA, 97.72 ± 6.15%; Xes+6-OHDA, 71.72 ± 5.01%. TUNEL-positive cells: Control, 6.68 ± 1.26%; 6-OHDA 50.76 ± 2.00%; 4-PBA+6-OHDA, 7.17 ± 0.30%; RY+6-OHDA, 13.02 ± 1.39%; Xes+6-OHDA, 52.32 ± 2.67%. Scale bar = 100 μm.

6-Hydroxydopamine is reported as a neurotoxin in cellular models of PD and has been proven to induce cell apoptosis. Here, we treated SN4741 cells with a range of 6-OHDA concentrations for 2 h. MTT assays showed that 60 μM 6-OHDA induced a significant decrease in cell viability compared with the control group (*P* < 0.01; **Figure 7A**, *n* = 10, one-way ANOVA). This effect was significantly reversed by 4-PBA or RY pretreatment (*P* < 0.01, respectively) but not by Xes pretreatment (**Figure 7B**, *n* = 10, one-way ANOVA). In addition, exclusive use of RY or Xes had no effect on cell viability. TUNEL staining (**Figures 7C,D**, *n* = 10, one-way ANOVA) also showed that 6-OHDA (60 μM) markedly increased cell apoptosis (*P* < 0.01), which was significantly reversed by 4-PBA or RY pretreatment (*P* < 0.01) but not by Xes pretreatment.

## Discussion

Parkinson’s disease is initially characterized by the loss of DA neurons in the SNc of the midbrain (Dauer and Przedborski, 2003). Studies have indicated that elevated and sustained ER stress is critically involved in the cellular Ca^2+^ overload and apoptosis seen in the 6-OHDA-induced PD model. Evidence also shows that the two main endogenous Ca^2+^ release channels, IP_3_Rs and RyRs, located on the ER surface play crucial roles in regulating Ca^2+^ homeostasis. However, the effects of these channels on the function and survival of DA neurons, as well as their roles in the pathology of PD, remain unknown. Thus, we investigated the roles of ER stress and the two endogenous Ca^2+^ release channels (IP_3_Rs and RyRs) in Ca^2+^ homeostasis, cellular excitability, and viability in a 6-OHDA-induced PD model. Our data are the first to demonstrate that RyRs rather than IP_3_Rs play a leading role in the response to 6-OHDA-mediated Ca^2+^ dyshomeostasis in DA neurons, contributing to a novel mechanism of neurotoxic injury in the 6-OHDA-induced PD model. Our findings also suggested a potential therapeutic strategy for PD by blocking the RyRs Ca^2+^ channels in the ER.

As an endogenous Ca^2+^ store, the ER is vital in intracellular Ca^2+^ regulation. ER stress is reported to disrupt Ca^2+^ homeostasis in response to multiple extracellular stimuli (Berridge et al., 2003). 6-OHDA, which is selectively taken up by DA neurons, is reported to induce ER stress responses (Ryu et al., 2002; Holtz and O’Malley, 2003), which are thought to be involved in the pathophysiology of PD in addition to oxidative stress. Our previous and present data strongly support that exposure to 6-OHDA can induce ER stress (**Figures 1A–D**) and indicate that 6-OHDA can significantly increase cytoplasmic Ca^2+^ levels in DA neurons (Qu et al., 2014) (**Figure 1E**) Additionally, we found that the above effect was significantly attenuated by the blockade of ER stress using 4-PBA (**Figure 1F**). Furthermore, we demonstrated that the effect of 6-OHDA on Ca^2+^ overload was sustained, even with immediate washout following 5 min of treatment in DA neurons (**Figures 2A,B**). These findings suggest that the sustained and irreversible Ca^2+^ dyshomeostasis in DA neurons induced by 6-OHDA may contribute to the pathophysiology of PD.

ER-mediated Ca^2+^ overload mainly occurs due to the dysfunction of the two Ca^2+^ release channels, RyRs and IP_3_Rs (Seo et al., 2015). Previous studies indicated that blockade of IP_3_Rs as well as RyRs strongly inhibited endothelin-1-induced Ca^2+^ release from intracellular stores in the rat pulmonary small artery (Kato et al., 2013). However, incubation with inhibitory RY does not affect ER Ca^2+^ content in primary hippocampal neurons (Adasme et al., 2015), in contrast to the significant Ca^2+^ depletion from the sarcoplasmic reticulum produced by inhibitory RY in skeletal muscle cells (Hwang et al., 1987). The existence of crosstalk between IP_3_Rs and RyRs is mentioned, but the impossibility of compensatory Ca^2+^ flux through one class of channel when the other type was blocked is also confirmed. However, how these channels influence Ca^2+^ in DA neurons remains unknown. Our data confirmed the roles of IP_3_Rs and RyRs in endogenous Ca^2+^ release and further demonstrated the different roles of IP_3_Rs and RyRs in the 6-OHDA-induced regulation of endogenous Ca^2+^ homeostasis in DA neurons. A RyRs blocker (RY) significantly alleviated the 6-OHDA-induced cytosolic Ca^2+^ increases (**Figures 3A,B**), whereas an IP_3_Rs blocker (Xes) had no observable effect (**Figures 3C,D**).

Dopaminergic neurons are characterized by autonomic excitability (clock-like, 2–4 Hz action potentials; Grace and Onn, 1989; Harris et al., 1989) and a selective dependence on Ca^2+^ channels (L-, N-, P/Q-, T and R-type Ca^2+^ channels) rather than Na^+^ channels for action potential generation (Grobaski et al., 1997; Bonci et al., 1998; Ping and Shepard, 1999). Studies have indicated that ER Ca^2+^ release activated plasma membrane K^+^ channels and contributed to the after hyperpolarizing potentials (AHPs), which then reduced neuronal activity in otic ganglion cells (Yoshizaki et al., 1995). Blocking RyRs/Ca^2+^-release channels with high doses of RY (100 μM) suppressed the rhythm in [Ca^2+^]_i_ and partially reduced the amplitude of the rhythm in the spike frequency of suprachiasmatic nucleus neurons in acute rat slices (Aguilar-Roblero et al., 2016).

Our study demonstrated that 6-OHDA induced sustained and irreversible changes in the excitability of DA neurons (**Figures 5A,B** and Supplementary Figures 1B, 2B). Moreover, the ER stress inhibitor 4-PBA significantly preserved the excitability of DA neurons in the SNc exposed to 6-OHDA (**Figures 4C,D** and Supplementary Figures 1A, 2A). Additionally, we are the first to confirm that inhibition of RyRs, but not IP_3_Rs, significantly preserved the excitability of DA neurons exposed to 6-OHDA (**Figures 5C–F** and Supplementary Figures 1C,D, 2C,D).

The Ca^2+^ content in the ER lumen as well as in the cytosol is very important for the function of mitochondrial and cellular viability (Michel et al., 2013). RyRs activation by caffeine and RY has been shown to induce apoptosis in hamster ovary cells via the depletion of intracellular Ca^2+^ (Pan et al., 2000), but RyRs inhibition for 24 h did not induce death in MIN6 cells (Luciani et al., 2009). Inhibiting IP_3_Rs, which disturbs constitutive Ca^2+^ transfer to the mitochondria, influences ATP production and leads to the activation of AMP-activated kinase (AMPK) and a subsequent increase in basal autophagic flux as a compensatory pro-survival response (Aguilar-Roblero et al., 2016). However, chronic IP_3_Rs inhibition with 2-aminoethoxydiphenyl borate and Xes decreased the survival of DA neurons in midbrain cultures (Missiaen et al., 1996). Our data showed that blockade of ER stress and Ca^2+^ overload protected against mitochondrial dysfunction induced by 6-OHDA (**Figures 6A–D**), which implies that the increase cytoplasmic Ca^2+^ levels induced by 6-OHDA may related to the dysfunction of mitochondrial (**Figure 6**) and pretreatment with the ER stress inhibitor 4-PBA protected DA neurons from 6-OHDA-induced apoptosis (**Figure 7**). Interestingly, IP_3_Rs inhibition failed to improve the viability of neurons exposed to 6-OHDA, whereas the RyRs inhibitor RY significantly alleviated the neurotoxic effects of 6-OHDA. Thus, these data suggest that RyRs Ca^2+^ channels in the ER may play a major role in 6-OHDA-induced Ca^2+^ dyshomeostasis and neurotoxic injury in PD.

## Conclusion

We have demonstrated for the first time that the Ca^2+^ dyshomeostasis induced by 6-OHDA in DA neurons is sustained and irreversible and that the IP_3_Rs and RyRs ER Ca^2+^ release channels play different roles in regulating endogenous Ca^2+^ homeostasis, neuronal excitability and viability in DA neurons. These results help clarify the pathophysiological mechanisms of PD and suggest a potential therapeutic strategy for PD by inhibiting the RyRs Ca^2+^ channels in the ER.

## Ethics Statement

This study was carried out in accordance with the recommendations of the ARRIVE guidelines, the U.K. Animals (Scientific Procedures) Act and the Institutional Animal Care and Use Committee at the Fourth Military Medical University.

## Author Contributions

GG and QY: Contributed to the conception or design of the work and drafting the work or revising it critically for important intellectual content. LH, YX, and DF: Substantial contributed to the conception or design of the work and acquisition, analysis, as well as interpretation of data for the work. RY and TN: Revised the work critically for important intellectual content. KT and GZ: Final proofreaded of the version to be published.

## Conflict of Interest Statement

The authors declare that the research was conducted in the absence of any commercial or financial relationships that could be construed as a potential conflict of interest.

## Abbreviations

4-PBA: 4-phenyl butyric acid
6-OHDA: 6-hydroxydopamine
ACSF: artificial cerebrospinal fluid
BAPTA-AM: 1,2-Bis(2-aminophenoxy)ethane-N,N,N’,N’-tetraacetic acid tetrakis (acetoxymethyl ester)
ER: endoplasmic reticulum
IP_3_Rs: inositol 1,4,5-triphosphate receptor
RY: ryanodine
RyRs: ryanodine receptors
SNc: substantia nigra pars compacta
Xes: xestospongin C
