# Supplementary material for: Blockade of RyRs in the ER Attenuates 6-OHDA-Induced Calcium Overload, Cellular Hypo-Excitability and Apoptosis in Dopaminergic Neurons
Source: Front Cell Neurosci. 2017 Mar 3;11:52. doi: 10.3389/fncel.2017.00052 (PMC5334509; doi:10.3389/fncel.2017.00052)
Supplement: Supplementary file 1 [file Data_Sheet_1.docx]

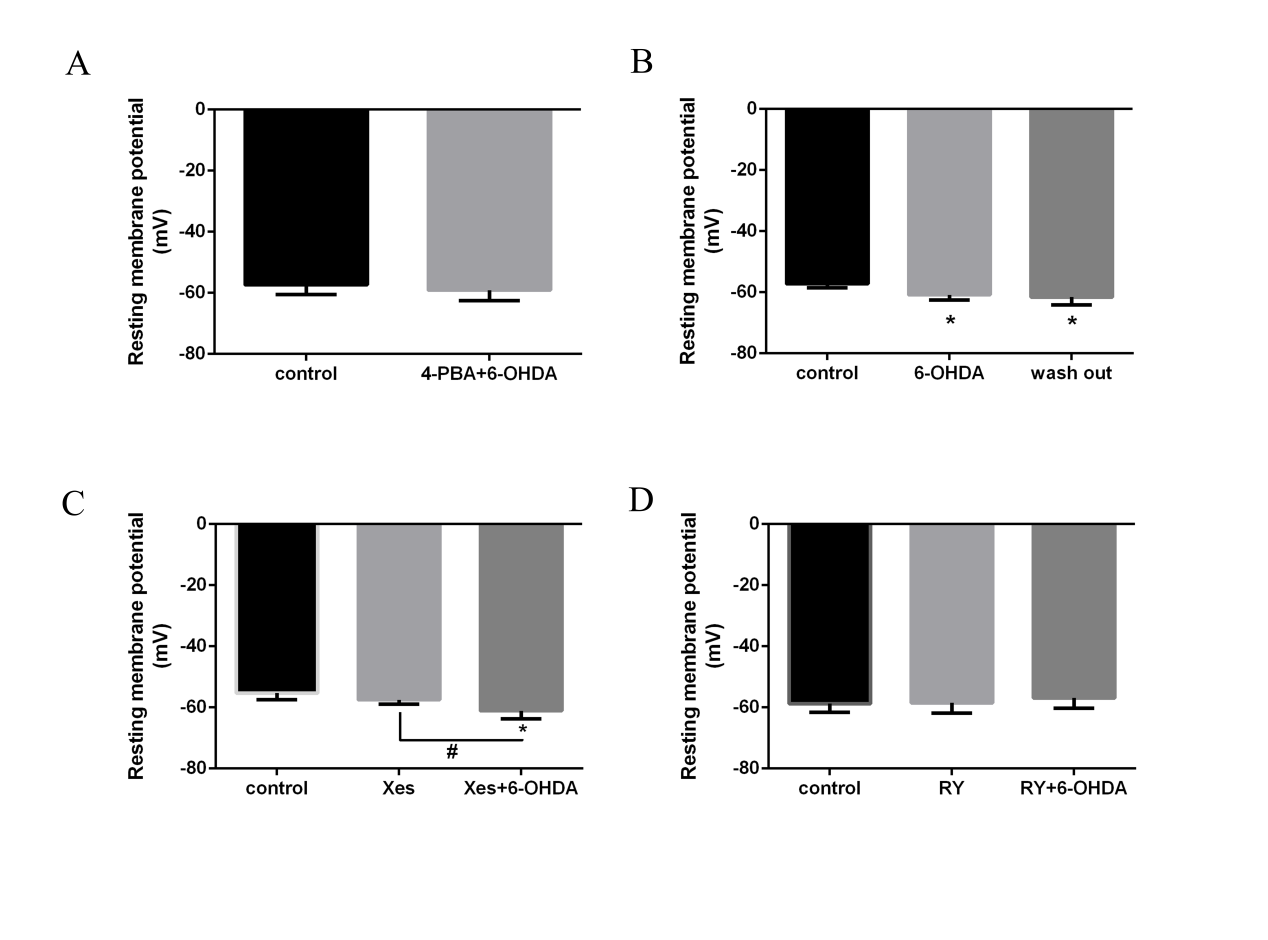


**Supplementary figure 1.** Resting membrane potential of DA neurons in SNc slices under different treatments. Resting membrane potential of DA neurons in SNc slices treated with 6-OHDA (1 μM) alone or with 4-PBA (2 mM) pretreatment for 20 min (A), treated with 6-OHDA (1 μM) and incubated in ACSF for another 10 min (B), pretreated with RY (100 μM) for 20 min followed by 6-OHDA (1 μM) (C), and pretreated with Xes (800 nM) for 20 min followed by 6-OHDA (1 μM) (D). Data are shown as mean ± SEM (n=10, one-way ANOVA or T-test). **P* < 0.05 *vs.* the control group，#P<0.05 in the chosen groups.


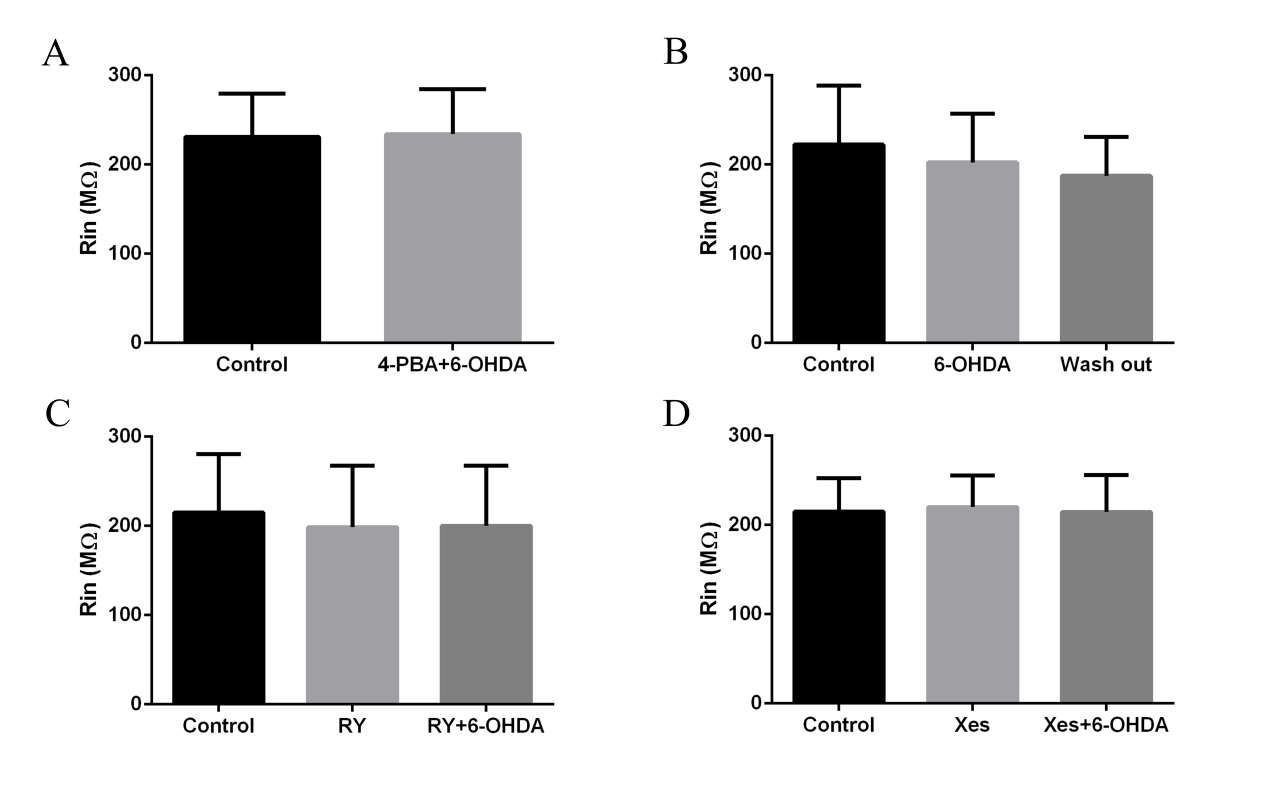


**Supplementary figure 2.** Input Resistance of DA neurons in SNc slices under different treatments. Input Resistance of DA neurons in SNc slices treated with 6-OHDA (1 μM) alone or with 4-PBA (2 mM) pretreatment for 20 min (A), treated with 6-OHDA (1 μM) and incubated in ACSF for another 10 min (B), pretreated with RY (100 μM) for 20 min followed by 6-OHDA (1 μM) (C), and pretreated with Xes (800 nM) for 20 min followed by 6-OHDA (1 μM) (D). Data are shown as mean ± SEM (n=10, one-way ANOVA or T-test).
